# Dog–Owner Relationship and Its Association with Social Cognition in French Bulldogs

**DOI:** 10.3390/ani15010017

**Published:** 2024-12-25

**Authors:** Lara Peterca, Elena Gobbo, Manja Zupan Šemrov

**Affiliations:** Department of Animal Science, Biotechnical Faculty, University of Ljubljana, Groblje 3, 1230 Domžale, Slovenia; elena.gobbo@bf.uni-lj.si (E.G.); manja.zupansemrov@bf.uni-lj.si (M.Z.Š.)

**Keywords:** French Bulldogs, brachycephalic dogs, cognition, MDORS, two-way object choice test, pointing gestures

## Abstract

Our understanding of social cognition in brachycephalic dog breeds is limited. This study focused specifically on French Bulldogs and hypothesized that a closer relationship between dog and owner would improve the dogs’ understanding of nonverbal cues, particularly pointing gestures. To investigate this, we tested twenty-six dogs and their owners in a two-way object choice test in which the familiar person pointed to the bowl. As understanding of pointing gestures is influenced by human–animal interaction, we used the Monash Dog Owner Relationship Scale (MDORS) to assess various aspects of the dog–owner relationship, including dog–owner interaction, emotional closeness, and negative aspects of dog ownership and measured salivary oxytocin levels. This study examined the dogs’ correct choices and their straight approach patterns to a reward bowl. Several factors influenced the two measures of social cognition, such as the age and sex of the dog, dog–owner interaction, emotional closeness, and the choice time (time it took the dog to reach the reward bowl), but also that neutered dogs and those with no training history showed better cognitive performance. We found a very high number of correct choices, which could be attributed to the owners’ high scores on the dog–owner interaction scale. Oxytocin showed no direct effect on these measures. To increase the generalizability of the results, we suggest future studies focus on more than one breed to capture the temporal development of the owner–dog relationship and social cognitive abilities.

## Simple Summary

This study explored how well French Bulldogs understand nonverbal communication, specifically whether having a close bond with their owner helps them interpret pointing gestures. Researchers worked with 26 French Bulldogs and their owners, testing whether the dogs could follow their owner’s pointing to find a treat hidden in a bowl. To measure the bond between each dog and their owner, they used a questionnaire and checked the dogs’ salivary oxytocin levels, a hormone linked to positive emotions. The results showed that factors like the dog’s age, gender, neuter status, and training experience affected how well they performed. Dogs with stronger bonds with their owners were generally better at understanding the pointing gestures, although oxytocin levels didn’t directly influence their choices. The authors suggest that future research should include more dog breeds to better understand how the owner–dog relationship influences social skills over time.

## 1. Introduction

Through domestication and long coexistence with humans, dogs have acquired cognitive abilities to recognize individual humans, remember past interactions, understand social relationships, and observe others [1]. Recent studies show that dogs exhibit advanced social cognitive skills in human interactions, such as word learning, selective attention, social learning [2], and understanding human gestures, which are crucial for their success in human environments [3,4,5]. Those skills have been shown to be influenced by their close relationship with humans [6,7].

Dogs approach their owners in emotional distress, use them as a secure base in new environments or stressful situations [8], and perform better in difficult tasks when owners are present [9]. They show better cue-following behavior [10,11], likely because dogs pay more attention to their owners than to strangers [12]. Owners provide protection [8], trust, security [13], and information [14], probably due to the closer/better relationship between a dog and a familiar person [15].

Recent research also indicates that a dog’s cognitive performance and emotional well-being are significantly influenced by the quality of their relationship with their owner. Important factors related to this relationship between the dog and its owner [16,17,18,19], gaze behavior [20], and cognitive performance [21] are associated with oxytocin. Oxytocin, a hormone associated with social behavior [22], positive emotional states [23], and animal welfare [22], is thought to be higher in dogs that have a closer relationship or feel more comfortable around their owner [18,24,25].

Other factors, for example breeds with specific morphology, can influence the level of dog’s social cognition and bond with humans. Gácsi et al. [26] found brachycephalic dog breeds performed better cognitively in a two-way object choice test than dolichocephalic dog breeds when an unfamiliar person pointed to the bowl. Since ganglion cells in brachycephalic dogs are more centrally located in the retina [27], it was hypothesized that these breeds respond best to stimuli in the central field (i.e., forward gaze) because they are not distracted by peripheral visual stimuli. However, Horschler et al. [28] did not found the correlation between morphological characteristics (e.g., head shape) and performance when the dogs were tested in a two-sided object choice test in which the owners pointed to the bowl. Ujfalussy et al. [29] found brachycephalic breeds were less successful in solving a cognitive task, but were much more attentive to humans than mesocephalic dogs, aiding in human-related task success. Bognár et al. [30] discovered that brachycephalic dogs looked longer towards human faces in the images shown, suggesting morphological influences on social interactions. Bognár et al. [31] confirmed that dogs with shorter heads made eye contact more quickly with unfamiliar people, indicating physical traits significantly impact social behaviors.

The French Bulldog is the most common and popular brachycephalic dog breed worldwide [32,33], having seen a significant rise in demand in recent years. However, this breed is also notable for its shortest life expectancy at birth and a predisposition to major health issues [34]. Another significant factor is that French Bulldog owners have higher attachment levels to their dogs compared to owners of dolichocephalic breeds [35]. Similarly, using the MDORS (Monash Dog Owner Relationship Scale) questionnaire [36], owners of brachycephalic dogs were found to generally form stronger relationships with their dogs, with higher scores on all three MDORS subscales compared to owners of dolichocephalic dogs [32]. This suggests that the physical characteristics of French Bulldogs, such as their appearance and ability to engage in communicative interactions with their owners, might contribute to the formation of these stronger bonds [37].

This study aimed to investigate the relationship between social cognition, i.e., understanding of the owner’s pointing gestures measured by a two-way object choice test, and the dog–owner relationship as measured by a standardized MDORS questionnaire and salivary oxytocin levels. Since owners of brachycephalic dogs generally have higher attachment scores, better results in the MDORS subscales were predicted to be associated with better performance in a social cognition task and higher oxytocin levels.

## 2. Materials and Methods

All procedures were performed in accordance with the legislation on the use of animals for scientific purposes in Slovenia and approved by the Animal Welfare Authority of the Department of Animal Science (reference: Z-02408/23). All owners signed an informed consent form and were given the right to withdraw from this study at any time without giving a reason.

### 2.1. Participants

Twenty-six French Bulldogs and their owners participated in this study. The dogs were of both sexes (16 females, 10 males; 10 neutered males and females), ranging in age from 1 to 9 years (mean = 3.92 years). All dogs were healthy, socialized, lived indoors, and were walked regularly. Sixteen dogs (61.5%) had no formal training history (e.g., training classes). Twenty-four dogs were obtained from a breeder, and two were bred at home. Owners accompanying the dogs during testing were predominantly female (*n* = 21, 81%) and between 20 and 51 years old (mean = 36.8 years). Five males (19%) were between 44 and 67 years old (mean = 53 years). Present owners were the dog’s primary attachment figure.

### 2.2. Procedure

This study took place in the summer of 2022 at the Biotechnical Faculty, Department of Animal Sciences, Slovenia, in an empty room unknown to the dogs. Prior to the start of the test, owners completed a demographic data questionnaire (owner age and sex, length of ownership, dog age and sex, neuter status, and training history) and the Monash Dog Owner Relationship Scale (MDORS) [36]. While owners completed the questionnaires and received instructions for the test, the dogs freely explored the testing room (5 m × 8 m) for approximately 15 min. In the next step, saliva was collected from the dogs, and this was immediately followed by the two-way object choice test [26]. None of the dogs had been previously trained for this particular test or participated in other cognitive testing.

### 2.3. Dog–Owner Relationship

The relationship between dog and owner was assessed with the MDORS, which consists of 28 items divided into 3 subscales. For the first subscale (dog–owner interaction), owners were asked to rate the frequency of a particular interaction with their dog on a 5-point scale (1 = less than once a month, 2 = once a month, 3 = once a week, 4 = several times a week, 5 = at least once a day). For the second subscale (emotional closeness), owners rated their agreement with statements about perceived emotional closeness related to social support, bonding, companionship, and unconditional love between dog and owner on a 5-point scale (1 = not true, 2 = mostly untrue, 3 = neither true nor false, 4 = mostly true, 5 = completely true). For the third subscale (perceived costs), owners were asked to rate the negative aspects of living with a dog on a 5-point scale (1 = not at all true, 2 = mostly untrue, 3 = neither true nor false, 4 = mostly true, 5 = completely true). The sum of all responses yielded the final score for each subscale, which was then used as continuous variables in the data analysis. Higher scores in the first two subscales and a lower score in the third subscale indicated a closer relationship between a dog and owner.

### 2.4. Salivary Oxytocin

To measure oxytocin concentration, saliva samples were collected 15 min after arrival at the testing room by the experimenter (*n* = 22) or by the owner if the dog was uncomfortable (*n* = 4). To avoid contamination of the samples, dogs were not allowed to drink or eat for at least half an hour before sampling. Commercially available absorbent cotton swabs in plastic tubes (Salivette^®^, Sarstedt, Rommelsdorft, Germany) were used for sample collection. The person collecting the sample wore latex gloves. Before saliva collection, the dog was shown the plastic tube and allowed to sniff it. The tube was then opened, and a cotton swab was removed and gently rotated on either side of the dog’s cheek pouch for 45 to 60 s (until saturation). The cotton swab was examined for visible contaminants, placed back in the plastic tube, and temporarily stored at −20 °C in the freezer.

Samples were taken to the laboratory for final storage and analysis. For the determination of oxytocin concentration, an immunological assay was used to quantify oxytocin in the samples, the Oxytocin ELISA kit from Enzo (New York, NY, USA). Analysis was performed according to the original manufacturer’s instructions and measurements were made directly in saliva. The sensitivity of the assay was 15.6 pg/mL, the intra-assay coefficient of variation was 10.2–13.3%, and the inter-assay coefficient of variation was 11.8–20.9%.

### 2.5. Social Cognition

Social cognition was measured using a two-way object choice test (modified from Gácsi et al. [26]), with the owner performing the pointing gestures, as in previous protocols [10,28,38]. Two aluminum bowls with rubber bottoms (11.5 cm in diameter and 4 cm high) were used as a hiding place for the reward. Except for five dogs that were allergic and whose owners brought their own food (diet pellets), small pieces of sausage were used as a reward.

**Figure 1:**
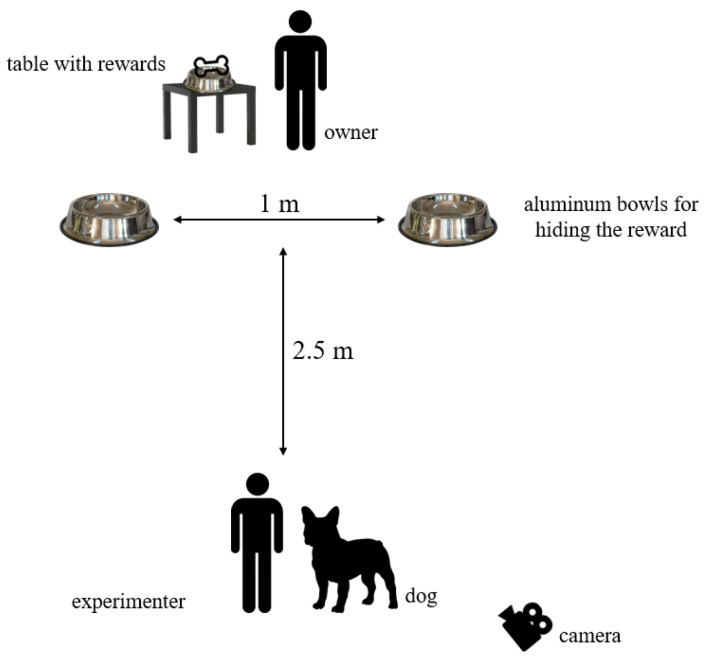
Schematic of the test area.

The owner sat on a chair 2.5 m from the experimenter who held the dog (Figure 1). On the left side of the chair was a table with rewards and bowls. Before the test, a demonstration trial was conducted. The owner placed both bowls on the floor at the same time at a distance of about 1 m from each other. When the owner placed the reward in the bowl so that the dog could see it, the experimenter had the dog go to the bowl and eat the reward. This was repeated twice, once for each bowl. In this way, both bowls had the smell of food and the dog was shown that the reward was in both bowls.

After the demonstration, the two-way choice test began. The owner placed the reward in a bowl while ensuring it was invisible to the dog by doing so when the dog was deliberately distracted, such as by turning the dog’s head or directing its gaze away from the bowl. Which bowl the owner placed the reward in (left or right) was instructed by the experimenter. In half of the repetitions, the reward was placed in the left bowl and the other half in the right bowl. The order was randomized, and a side could only be repeated twice in a row and not immediately at the beginning of the experiment. After the reward was placed in one bowl, the owner placed both bowls on the floor at the same time. If the dog did not focus on the owner right away, the owner called the dog by name to obtain the dog’s attention. When attention was obtained, the owner pointed to the reward bowl with an extended index finger. The gesture was firm and lasted approximately one second, with the index finger about 20 cm from the bowl. Before and after pointing, the owners crossed their arms in front of their chests. The experimenter held the dog and released it after the owner’s pointing gesture. The dog was held by the experimenter during the consistent repetitions of pointing. The bowl that the dog approached first or within 10 cm was considered the chosen one. The dog could only eat the reward if the correct bowl was chosen, otherwise the reward bowl was taken away. The bowl was also taken away if the dog chose the correct bowl but did not eat the reward (*n* = 1). The gesture procedure was repeated 20 times without interruption. Between individual dogs, the bowls were thoroughly cleaned with detergent and hot water to remove any residual food odors, to ensure that the scent of other dogs did not influence the choices. The camera (Nikon D5300 digital camera, Nikon Corporation, Tokyo, Japan) was placed behind the experimenter so that all the dog’s movements and the owner’s pointing gestures were recorded. One person observed and scored all of the videos.

**Figure 2:**
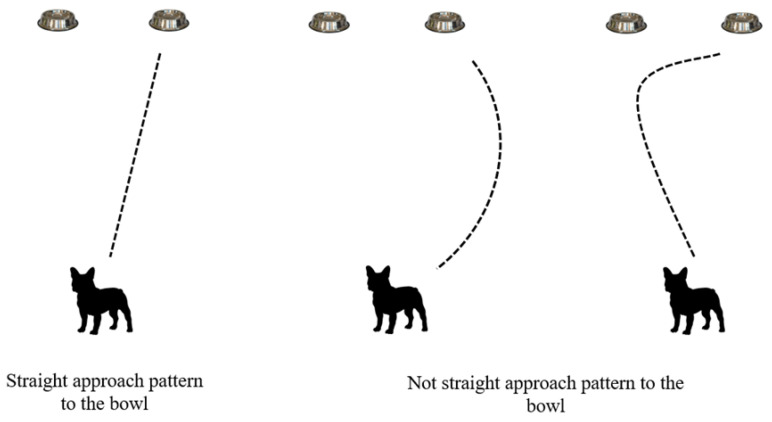
Categorization of the patterns of approach to the reward bowl.

The first parameter observed was the number of correct choices of the reward bowl by the dog based on a pointing gesture by the owner (correct choices). The next parameter was the pattern of approach to the rewarded bowl (approach pattern). The pattern was categorized as straight if the dog approached the selected bowl in a straight line, or not straight if the dog approached the bowl in a crooked manner or turned toward the other bowl at the last moment (Figure 2). The time it took the dog to reach the reward bowl (choice time) was the third observed measurement. Observed from the videos, choice time (sec) began when the dog was released by the experimenter and ended when the dog selected the bowl (even if the selection was incorrect). The choice time is later represented as the sum of all 20 repetitions. There was one dog that did not leave the starting position within 5 s. In this case, the owner had to regain the dog’s attention by calling the dog’s name and then repeating the pointing gesture on three more repetitions. The dog was still being held by the experimenter. For this dog, only the time in which the dog successfully made the choice was used. The time was measured with a stopwatch.

### 2.6. Data Analysis

Statistical analysis was performed using SAS/STAT software, version 9.4 of the SAS System for Windows, with an individual dog as the statistical unit. The differences in the number of correct choices made by an individual and the approach pattern data were calculated on the basis of a Poisson distribution. For the latter data, only the number of straight approach patterns was included in the analysis. Out of the 20 repetitions for each dog, only those classified as straight approach patterns were analyzed, as their occurrence was notably high (mean = 16.46). Despite the fact that one dog exclusively chose the right bowl, the data were left in the statistical analysis, as was the case in previous studies [39,40].

We used the GLIMMIX procedure, which fits and analyzes generalized linear mixed models with random effects for data that may not be normally distributed, and tested the significance of the effects by an X2 test. The following fixed effects were included in the model: sex of dog (female, male), neuter status (neutered and intact), training history (trained dogs and untrained dogs), and gender of owner (woman, man). In addition to fixed effects, we also tested continuous variables: age of owner, age of dog, dog–owner interaction, emotional closeness, perceived costs, choice time (sec), and oxytocin level in saliva (pg/mL). The dog’s age and the length of ownership proved to be too similar, so only the dog’s age was included in the model. The final statistical models were developed as follows:

Formula for number of correct choices:

$$y_{ijkl\;}=µ+S_{i}+T_{j}+b_{I}(x_{ijk}-x)+b_{II}(x_{ijk}-x)+b_{III}\;(x_{ijk}-x)+e_{ijkl}$$

$y_{ijkl}\;$= observed characteristic, correct choices
µ = mean value
$S_{i}$ = qualitative systematic effect of sex of the dog, *i* = 1, 2
$T_{j}$ = qualitative systematic impact of training history, *j* = 1, 2
$b_{I}$ = regression coefficient for the dog–owner interaction
$b_{II}$ = regression coefficient for the emotional closeness
$b_{III}=$ regression coefficient for the choice time
$e_{ijkl}$ = residue

Formula for approach pattern to the bowl:

$$y_{ijk}=µ+N_{i}+b_{I}(x_{ijk}-x)+b_{II}(x_{ijk}-x)+e_{ijk}$$

$y_{ijk}=$ observed characteristic, approach pattern to the bowl
µ = mean value
$N_{i}=$ qualitative systematic effect of neuter status, *i* = 1, 2
$b_{I}\;$= regression coefficient for the age of dog
$b_{II}=$ regression coefficient for the dog–owner interaction
$e_{ijk}=$ residue

In developing a model, a fixed effect that did not have a significant effect was removed from the model, and the data set was reanalyzed. Statistical significance was assumed when *p* ≤ 0.05, trending toward significance at *p* ≤ 0.10. Only significant values are presented in this article. The final model for correct choice included the sex of the dog, training history, dog–owner interaction, emotional closeness, and choice time. The final model for the approach pattern to the bowl included neuter status, age of the dog, and dog–owner interaction. To test the correlation between different parameters but not correct choices, a correlation analysis was performed using Kendal’s correlation coefficient. In view of the small sample size and the large number of variables, both models were adjusted using the F-test. The F-values are given in the text. The generalized chi-square was 91.27 for the first model developed and 83.05 for the second model.

## 3. Results

### 3.1. Dog–Owner Relationship and Cognitive Data

For MDORS (Table 1), the highest score for dog–owner interaction was 43 and the lowest was 32 (mean ± SD = 36.38 ± 2.70). For emotional closeness, the highest score was reported as 50 and the lowest as 38 (45.19 ± 3.75). For perceived costs, the highest score was 32 and the lowest was 9 (14.58 ± 5.97). Table 1 also shows descriptive results for the two-way object choice test, which involved correct choices. The lowest score was recorded as 10 and the highest (achieved by two dogs) as 20. The average number of correct choices was 16.12 ± 3.14. At the individual level, one dog exclusively chose only the right bowl. The fastest choice time was 35 s and the slowest was 149 (73.5 ± 27.75). The maximum number of straight approach patterns (20) was achieved by six dogs and the minimum number of straight approach patterns (11) was achieved by only one dog (16.46 ± 2.86). Oxytocin levels ranged from 15.6 to 217 pg/mL (87.07 ± 53.11 pg/mL) and had no effect on the two-way object test (Table 2).

The number of correct choices was significantly influenced by five factors, namely the sex of the dog, the training history, dog–owner interaction, the emotional closeness, and choice time (Table 2). The number of correct choices was higher in males (mean ± SD = 16.8 ± 3.2) than in females (15.4 ± 3.2) and higher in dogs with no training history (i.e., untrained dogs; 17.1 ± 2.6) than in dogs with training history (i.e., trained dogs; 16.1 ± 3.7). We also found that the number of correct choices increased with increasing dog–owner interaction but decreased with increasing emotional closeness. The number of correct choices also decreased as the dog spent more time making choices.

Three factors, the age of the dog, neuter status (neutered male and female dogs = 4.3 ± 2.7 years, un-neutered male and female dogs = 3.7 ± 2.6 years), and dog–owner interaction, influenced the approach pattern to the bowl (Table 3). The number of straight approaches to the bowl increased with increasing age of the dog and also with increasing interaction between dog and owner. In addition, we found that the higher number of straight approaches to the bowl was influenced by neuter status, with neutered dogs performing better (mean ± SD = 17.6 ± 2.6) than un-neutered dogs (mean ± SD = 14.6 ± 2.5).

### 3.2. Correlation Analysis Data

**Figure 3:**
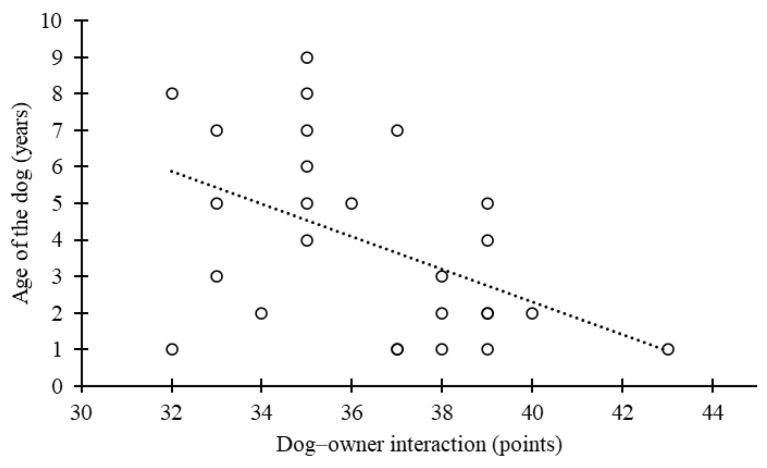
Relationship between the age of the dog and dog–owner interaction (a higher points score means a higher level of interaction).

**Figure 4:**
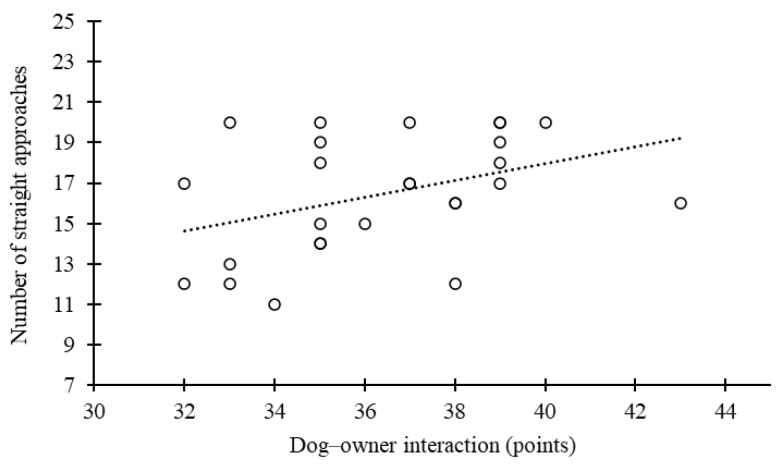
Relationship between dog–owner interaction (a higher points score means a higher level of interaction) and number of straight approaches to the bowl.

Correlation analysis showed that dog–owner interaction was negatively correlated with the age of the dog (r = −0.48, *p* = 0.01) (Figure 3) and positively correlated with a straight approach pattern (r = 0.39, *p* = 0.05) (Figure 4). No other correlations were found, including between oxytocin levels and the MDORS subscales.

## 4. Discussion

This study investigated the relationship between dog–owner interaction and the social cognition of French Bulldogs, a brachycephalic breed that has not been extensively researched in the field of social cognition. We hypothesized that dogs with a better understanding of their owner’s pointing gestures would also have a stronger relationship with their owner, as indicated by higher MDORS interaction and emotional closeness scores, lower perceived cost scores, and higher salivary oxytocin levels. The hypothesis was partially confirmed: dogs whose owners had higher interaction scores showed better cognitive performance, but there was no effect of oxytocin on social cognition.

As expected, the MDORS subscales showed low variability, reflecting generally higher attachment scores among brachycephalic dog owners [35]. High scores on the dog–owner interaction subscale suggest a close relationship, consistent with other studies on brachycephalic dogs [32,35]. Dogs with more frequent interaction with their owners may better understand pointing gestures due to spending more time observing and interpreting their owner’s behavior [5,7]. Research indicates that the quality of the dog–owner relationship is crucial for a dog’s social learning abilities, particularly in understanding human gestures like pointing [41,42]. The frequency and quality of interactions significantly contribute to developing these social–cognitive skills in dogs. Our finding that dog–owner interaction influences performance in the two-way choice test aligns with Langner et al. [6], who found dogs responded better to familiar voices in the test. Better performance was indicated by more correct choices and straighter approaches to the bowl, suggesting a better understanding of pointing gestures. This might be because these dogs are less independent [43] and spend more time with their owners [44]. Additionally, it is important to consider that the popularity of French Bulldogs may be partly attributable to their distinctive appearance, which resembles that of human infants, including large eyes and a flat face, and facilitates human–animal communication and bonding [45,46]. This resemblance to human infant features is known to elicit nurturing behaviors in humans, facilitating stronger emotional bonds and more frequent social interactions between dogs and their owners. Consequently, this appearance is likely to promote prolonged eye contact and emotional closeness between owners and their dogs, which in turn increases the effectiveness of communicative interactions. These enhanced interactions could help to explain why French Bulldogs show improved performance on socio-cognitive tests. The frequent, high-quality interactions between these dogs and their owners provide more opportunities for interpreting human gestures, contributing to better social cognition [47].

Contrary to expectations, greater emotional closeness scores were associated with fewer correct choices in the two-way choice test. This aligns with Rehn et al. [43], suggesting that while owners may feel a strong emotional attachment to their dogs, the feeling might not be reciprocated equally by the dogs. Interestingly, the perceived cost of dog ownership did not significantly affect social cognition. Similarly, Bouma et al. [48], indicated that owners of breeds with known health issues, such as French Bulldogs, anticipate high veterinary costs and do not view them as prohibitive or burdensome. Also, Packer et al. [32] found that 65% of French Bulldog owners expect high veterinary costs, suggesting they are prepared for these financial implications as part of their commitment to the breed. In addition to their appearance, French Bulldogs are widely regarded as suitable companion animals due to their positive behavioral traits, including loyalty, an affectionate nature, playfulness, and ease of training. These qualities render them particularly well-suited to families with children. These traits also justify the decision to focus this study exclusively on French Bulldogs, as their behavioral and emotional profile differs from that of other dog breeds [46].

The sex of the dog also influences cognitive performance. Recent studies [49,50] and our research show that male dogs perform better in some cognitive tests compared to females. However, other research suggests females excel in a number of social cognition tasks [51,52,53] and physical cognition task [54]. Some studies find no sex differences in social [29] or spatial cognition [55,56]. The reasons for these differences are unclear, but hormonal levels, genes on sex chromosomes, and personality traits may play a role. Males often show higher courage scores in unfamiliar situations, such as encounters with new experimenters, rooms, and tasks, which might influence cognitive performance [57,58].

Unlike some studies that reported no effect of training on cognitive test performance [10,39,52], our study found that untrained dogs performed better in cognitive tests. Gácsi et al. [39] suggested that dogs’ ability to rely on human pointing gestures remains robust and relatively resistant to environmental influences after 2–4 months of age, implying that other abilities may underlie success in cognitive tests [59,60]. However, some studies, including Marshall-Pescini et al. [61], reported that training improves dogs’ behavior in socio-cognitive tasks [13,62,63]. The unexpected outcome of our study may partly reflect limitations related to sampling. Although this study included 26 dogs, it was limited to a single breed. In contrast, other studies examined up to 25 dogs from a variety of mixed breeds [10,38,64,65], which might have contributed to greater data variability.

In the two-way choice test, quicker decision times were linked to better performance. Positive associations between choice time, approach, and dog–owner interaction suggest that dogs spending more quality time with their owners recognized the pointing gesture immediately and chose the correct bowl without hesitation. Greater food motivation might also explain this, as dogs with better cognition anticipated a reward and thus performed better [66], but it is difficult to consider it in our study since we did not test the level of food motivation prior to testing. Bhattacharjee et al. [67] found dogs following pointing gestures had greater accuracy, and Schmidjell et al. [68] showed dogs predominantly followed pointing gestures over subtle owner cues, indicating a strong innate understanding.

Neuter status influenced approach patterns, with neutered dogs (males and females) more frequently approaching the bowl in a straight line. Comparisons with other studies are challenging due to limited research on neutering effects in two-way choice tests. However, Scandurra et al. [69] noted socio-cognitive decline and reduced attention in unsterilized females compared to sterilized ones. Age also significantly influenced approach patterns; older dogs, likely due to prolonged exposure to owner gestures, approached in a straight line more often. Persson et al. [70] found that human-directed social behavior, including eye contact, increased with age, suggesting that age plays a crucial role in social cognition and attachment, potentially explaining why older neutered dogs (males and females) performed better.

Contrary to our initial expectations for typical performance from a brachycephalic breed in the two-way object choice test, our cognitive results were unexpectedly high. Previous studies [3,5,6,26,39] did not report such high average number of correct choices, highlighting a notable difference in outcomes. This remarkable performance led to low variability in our data, likely influenced by the dogs’ heightened visual acuity or the owners’ strong attachment scores.

In contrast to studies on dogs [39], horses [71], and goats [40], we observed only one instance of side bias during the test. In this case, a dog consistently chose the right bowl, possibly due to a reduced tendency to follow gestures and a preference for maintaining the reward in the same location (“win–stay strategy”) [72]. Alternatively, as suggested by Gácsi et al. [39], the decision rule for subsequent trials might have been influenced by the initial success; the reward pointed out by the owner during the first trial was in the right bowl, leading the dog to continue selecting it throughout the test. Kis et al. [73] reported that side bias varies with the sex and age of the dog and is stable over time [74]. Older dogs and females tend to develop side preferences more quickly, which may explain the observed side bias in our study’s 8-year-old female dog. Brain lateralization could also play a role, with the left hemisphere associated with positive emotions [75,76] and approach behaviors [64]. Consequently, we can infer that our dog positively associated with the right bowl.

Our study found a negative correlation between human–dog interaction and the dog’s age, with older dogs experiencing fewer interactions with their owners. This supports previous findings that interactions are more frequent in younger dogs, who require more time and attention for training and developmental care [77].

Surprisingly, we found no association between oxytocin concentration, cognitive performance, and/or dog–owner relationship, despite oxytocin’s known role in modulating these factors [19,20,21]. This may be due to only one saliva sample per dog or sampling error, such as restlessness during sampling or previous water intake, despite owners being instructed not to give their dogs a drink at least 30 min before the test. Another factor could be the nature of the saliva, which varies under different nervous system controls [78], affecting oxytocin levels or perhaps because the average values were lower, as values above 200 are typically reported in dogs [79]. Caution is needed when interpreting our oxytocin results, as some authors report an oversimplified view of oxytocin’s role in social relationships, including the dog–human relationship [13,16,80].

## 5. Limitations and Conclusions

In conclusion, our results highlight the nuanced interplay of various factors that influence dogs’ cognitive and behavioral responses in decision-making tasks. Our study’s unexpectedly high social–cognitive results, characterized by a substantial number of successful choices in the two-way object choice test in a brachycephalic breed, also prompt several critical considerations for future research. First, our findings underscore the potential influence of attachment scores on research outcomes and data variability. Investigating this factor in parallel with behavioral responses during social cognitive tests or other cognitive contexts could provide valuable insights. Secondly, social cognition research in dogs remains scarce, particularly studies involving larger samples of a single breed. Such studies can bring valuable insight since we can, to some extent, control for potentially important factors such as genetics, the general role of the breed (e.g., French Bulldogs are mostly companion dogs that don’t work), athletic/locomotor abilities, and morphology. This scarcity contributes to inconsistent and challenging-to-interpret data. Also, since the level or type of training can influence performance during testing and attachment, simply having data about training or no training might not be sufficient, and this parameter should be explored more thoroughly. Future efforts should prioritize diverse samples to enhance the robustness of findings. Regarding physiological parameters, such as saliva oxytocin, rigorous standardization and controlled conditions before sample collection are crucial. Only under such circumstances can we confidently consider these measures as reliable indicators of dog social cognition. Although we instructed dog owners not to feed their dogs for an hour and to avoid drinking for at least 30 min before sampling, we lacked control over compliance. This oversight could potentially impact emotional attachments and relationship dynamics.

The exclusive focus of this study on French Bulldogs may limit the applicability of the results on a broader scale, as the breed specificity may not adequately represent the broader dog population, limiting the generalizability of the results. In addition, the lack of a control group, such as dogs of other breeds or with different owner profiles, limits the comparative analysis of breed-specific behaviors and owner–dog relationship dynamics. Finally, the cross-sectional design of this study does not capture the temporal development of the dog–owner relationship and the development of social cognitive skills, so a longitudinal approach is needed for a more comprehensive understanding of these dynamics over time. We suggest that these limitations be addressed in future studies.

## Figures and Tables

**Table 1:** Descriptive results of the two-way object choice test and the Monash Dog Owner Relationship Scale.

Dog	Correct Choices ^1^	Choice Time (s)	Straight Approach Pattern ^2^	Dog–Owner Interaction ^3^	Emotional Closeness ^4^	Perceived Costs ^5^	Dog’s Age	Dog’s Sex	Neuter Status	Training History
A1	20	74	20	37	49	12	7	M	intact	trained
A2	10	59	20	33	43	16	7	F	intact	trained
A3	12	69	12	33	43	16	5	F	neutered	trained
A4	12	138	13	33	43	16	3	M	intact	trained
A5	18	77	17	37	46	12	1	M	intact	untrained
A6	19	119	16	43	39	9	1	F	neutered	untrained
A7	18	71	17	37	47	13	1	F	intact	untrained
A8	11	97	14	35	47	14	8	M	neutered	trained
A9	17	63	15	36	44	22	5	F	neutered	untrained
A10	16	43	15	35	47	9	6	F	intact	untrained
A11	19	37	18	35	47	9	4	F	intact	untrained
A12	13	42	14	35	49	32	5	F	neutered	untrained
A13	11	149	19	35	49	32	9	F	neutered	untrained
A14	19	87	19	39	38	9	5	F	intact	untrained
A15	19	73	20	39	38	9	2	F	intact	untrained
A16	20	80	20	39	38	9	2	F	intact	untrained
A17	14	91	12	38	49	12	2	M	intact	trained
A18	16	52	16	38	50	16	3	F	neutered	trained
A19	14	35	12	32	43	15	1	F	neutered	untrained
A20	18	44	11	34	44	15	2	M	neutered	untrained
A21	15	57	18	39	49	14	1	F	intact	untrained
A22	13	63	17	39	49	14	4	F	neutered	trained
A23	18	78	16	38	48	12	1	M	intact	untrained
A24	19	62	20	40	48	16	2	M	intact	trained
A25	19	80	20	35	46	17	7	M	intact	untrained
A26	19	71	17	32	42	9	8	M	intact	trained

^1^ Maximum number of correct choices = 20; ^2^ maximum score for straight approach pattern = 20; ^3^ maximum score for dog–owner interaction = 45; ^4^ maximum score for emotional closeness = 50; ^5^ maximum score for perceived costs = 45.

**Table 2:** Results of the effects on correct choices in the two-way object choice test.

	F-Value	*p*-Value	Estimates
Dog’s sex (females vs. males)	12.95	0.018	−3.47
Dog’s age	1.38	0.26	0.25
Neuter status (neutered vs. intact)	0.01	0.92	−0.12
Training history (trained vs. untrained)	5.59	0.03	−2.24
Gender of owner	0.74	0.41	−1.51
Owner’s age	2.72	0.12	−0.13
Dog–owner interaction	13.70	0.001	0.60
Emotional closeness	6.46	0.02	−0.30
Perceived costs	1.23	0.29	−0.12
Choice time	8.62	0.008	−0.05
Oxytocin level	1.15	0.30	0.01

Values for significant effects are shown from the final model; all other values are from the previous steps.

**Table 3:** Results of the effects on pattern of approach to the reward bowl in the two-way object choice test.

	F-Value	*p*-Value	Estimates
Dog’s sex (females vs. males)	0.51	0.49	0.62
Dog’s age	13.08	0.002	0.69
Neuter status (neutered vs. intact)	14.52	0.001	−3.01
Training history (trained vs. untrained)	0.39	0.54	0.68
Gender of owner	1.38	0.26	−1.96
Owner’s age	0.12	0.74	−0.03
Dog–owner interaction	15.31	0.0007	0.63
Emotional closeness	1.40	0.26	−0.22
Perceived costs	2.89	0.11	0.18
Choice time	1.54	0.23	−0.02
Oxytocin level	0.18	0.68	0.004

Values for significant effects are shown from the final model; all other values are from the previous steps.

## Data Availability

The dataset used for the analysis is included as Supplementary Materials.

## Supplementary Materials

The following supporting information can be downloaded at https://www.mdpi.com/article/10.3390/ani15010017/s1, Table S1: Dataset.

## References

[1] Seyfarth R.M., Cheney D.L. (2015). Social cognition. Anim. Behav..

[2] Range F., Virányi Z. (2015). Tracking the evolutionary origins of dog-human cooperation: The canine cooperation hypothesis. Front. Psychol..

[3] Pelgrim M.H., He I.X., Lee K., Pabari F., Tellex S., Nguyen T., Buchsbaum D. (2024). Find it like a dog: Using gesture to improve object search. Cogsci.

[4] Salamon A., Uccheddu S., Csepregi M., Miklósi Á., Gácsi M. (2023). Dogs outperform cats both in their testability and relying on human pointing gestures: A comparative study. Sci. Rep..

[5] Udell M.A.R. (2018). A new approach to understanding canine social cognition. Learn. Behav..

[6] Langner L., Žakelj S., Bolló H., Topál J., Kis A. (2023). The influence of voice familiarity and linguistic content on dogs’ ability to follow human voice direction. Sci. Rep..

[7] Marshall-Pescini S., Virányi Z., Kubinyi E., Range F. (2017). Motivational factors underlying problem solving: Comparing wolf and dog puppies’ explorative and neophobic behaviors at 5, 6, and 8 weeks of age. Front. Psychol..

[8] Gácsi M., Maros K., Sernkvist S., Farago T., Miklósi Á. (2013). Human analogue safe haven effect of the owner: Behavioural and heart rate response to stressful social stimuli in dogs. PLoS ONE.

[9] Horn L., Huber L., Range F. (2013). The importance of the secure base effect for domestic dogs: Evidence from a manipulative problem-solving task. PLoS ONE.

[10] Cunningham C.L., Ramos M.F. (2014). Effect of training and familiarity on responsiveness to human cues in domestic dogs (*Canis familiaris*). Anim. Cogn..

[11] Elgier A.M., Jakovcevic A., Mustaca A.E., Bentosela M. (2009). Learning and owner-stranger effects on interspecific communication in domestic dogs (*Canis familiaris*). Behav. Process..

[12] Mongillo P., Bono G., Regolin L., Marinelli L. (2010). Selective attention to humans in companion dogs, *Canis familiaris*. Anim. Behav..

[13] Marshall-Pescini S., Schaebs F.S., Gaugg A., Meinert A., Deschner T., Range F. (2019). The role of oxytocin in the dog–owner relationship. Animals.

[14] Merola I., Prato-Previde E., Lazzaroni M., Marshall-Pescini S. (2014). Dogs’ comprehension of referential emotional expression: Familiar people and familiar emotions are easier. Anim. Cogn..

[15] Horn L., Range F., Huber L. (2013). Dogs’ attention towards humans depends on their relationship, not only on social familiarity. Anim. Cogn..

[16] Buttner A.P. (2016). Neurobiological underpinnings of dogs’ human-like social competence: How interactions between stress response systems and oxytocin mediate dogs’ social skills. Neurosci. Biobehav. Rev..

[17] Kis A., Ciobica A., Topál J. (2017). The effect of oxytocin on human-directed social behaviour in dogs (*Canis familiaris*). Horm. Behav..

[18] MacLean E.L., Gesquiere L.R., Gee N.R., Levy K., Martin W.L., Carter C.S. (2017). Effects of affiliative human–animal interaction on dog salivary and plasma oxytocin and vasopressin. Front. Psychol..

[19] Nagasawa M., Mitsui S., En S., Ohtani N., Ohta M., Sakuma Y., Onaka T., Mogi K., Kikusui T. (2015). Oxytocin-gaze positive loop and the coevolution of human-dog bonds. Science.

[20] Dzik M.V., Cavalli C.M., Barrera G.C., Bentosela M. (2020). Oxytocin effects on gazing at the human face in retriever dogs. Behav. Process..

[21] Oliva J.L., Mengoli M., Mendonça T., Cozzi A., Pageat P., Chabaud C., Teruel E., Lafont-Lecuelle C., Bienboire-Frosini C. (2019). Working smarter not harder: Oxytocin increases domestic dogs’ (*Canis familiaris*) accuracy, but not attempts, on an object choice task. Front. Psychol..

[22] Rault J.L., van den Munkhof M., Buisman-Pijlman F.T.A. (2017). Oxytocin as an indicator of psychological and social well-being in domesticated animals: A critical review. Front. Psychol..

[23] Mitsui S., Yamamoto M., Nagasawa M., Mogi K., Kikusui T., Ohtani N., Ohta M. (2011). Urinary oxytocin as a noninvasive biomarker of positive emotion in dogs. Horm. Behav..

[24] Handlin L., Nilsson A., Ejdebäck M., Hydbring-Sandberg E., Uvnäs-Moberg K. (2012). Associations between the psychological characteristics of the human-dog relationship and oxytocin and cortisol levels. Anthrozoos.

[25] Ogi A., Mariti C., Baragli P., Sergi V., Gazzano A. (2020). Effects of stroking on salivary oxytocin and cortisol in guide dogs: Preliminary results. Animals.

[26] Gácsi M., McGreevy P., Kara E., Miklósi Á. (2009). The effects of selection for cooperation and attention in dogs. Behav. Brain Funct..

[27] McGreevy P., Grassi T.D., Harman A.M. (2004). A strong correlation exists between the distribution of retinal ganglion cells and nose length in the dog. Brain Behav. Evol..

[28] Horschler D.J., Hare B., Call J., Kaminski J., Miklósi Á., MacLean E.L. (2019). Absolute brain size predicts dog breed differences in executive function. Anim. Cogn..

[29] Ujfalussy D., Bognár Z., Molnár M., Miklósi Á., Kubiniy E. (2023). The difference between two brachycephalic and one mesocephalic dog breeds’ problem-solving performance suggests evidence for paedomorphism in behaviour. Sci. Rep..

[30] Bognár Z., Iotchev I.B., Kubinyi E. (2018). Sex, skull length, breed, and age predict how dogs look at faces of humans and conspecifics. Anim. Cogn..

[31] Bognár Z., Szabó D., Deés A., Kubinyi E. (2021). Shorter headed dogs, visually cooperative breeds, younger and playful dogs form eye contact faster with an unfamiliar human. Sci. Rep..

[32] Packer R.M.A., O’Neill D.G., Fletcher F., Farnworth M.J. (2019). Great expectations, inconvenient truths, and the paradoxes of the dog-owner relationship for owners of brachycephalic dogs. PLoS ONE.

[33] Ravn-Mølby E.M., Sindahl L., Nielsen S.S., Bruun C.S., Sandøe P., Fredholm M., Loor J.J. (2019). Breeding French bulldogs so that they breathe well- A long way to go. PLoS ONE.

[34] Teng K.T., Brodbelt D.C., Pegram C., Church D.B., O’Neill D.G. (2022). Life tables of annual life expectancy and mortality for companion dogs in the United Kingdom. Sci. Rep..

[35] Sandøe P., Kondrup S.V., Bennett P.C., Forkman B., Meyer I., Proschowsky H.F., Serpell J.A., Lund T.B., Rosenfeld C.S. (2017). Why do people buy dogs with potential welfare problems related to extreme conformation and inherited disease? A representative study of Danish owners of four small dog breeds. PLoS ONE.

[36] Dwyer F., Bennett P.C., Coleman G.J. (2006). Development of the monash dog owner relationship scale (MDORS). Anthrozoos.

[37] Packer R.M. (2021). Flat-faced fandom: Why do people love brachycephalic dogs and keep coming back for more?. Front. Vet. Sci..

[38] Elgier A.M., Jakovcevic A., Barrera G., Mustaca A.E., Bentosela M. (2009). Communication between domestic dogs (*Canis familiaris*) and humans: Dogs are good learners. Behav. Process..

[39] Gácsi M., Kara E., Belényi B., Topál J., Miklósi Á. (2009). The effect of development and individual differences in pointing comprehension of dogs. Anim. Cogn..

[40] Kaminski J., Riedel J., Call J., Tomasello M. (2005). Domestic goats (*Capra hircus*) follow gaze direction and use some social cues in an object choice task. Anim. Behav..

[41] McCartney M.J., Leavens D.A. (2024). The role of life history and familiarity in performance of working and non-working dogs (*Canis lupus familiaris*) in a point-following task. Animals.

[42] Pongrácz P., Rieger G., Vékony K. (2021). Grumpy dogs are smart learners—The association between dog–owner relationship and dogs’ performance in a social learning task. Animals.

[43] Rehn T., Lindholm U., Keeling L., Forkman B. (2014). I like my dog, does my dog like me?. Appl. Anim. Behav. Sci..

[44] Wedl M., Schöberl I., Bauer B., Day J., Kotrschal K. (2010). Relational factors affecting dog social attraction to human partners. Interact. Stud..

[45] Thorn P., Howell T.J., Brown C., Bennett P.C. (2015). The canine cuteness effect: Owner-perceived cuteness as a predictor of human–dog relationship quality. Anthrozoös.

[46] Packer R.M., O’Neill D.G., Fletcher F., Farnworth M.J. (2020). Come for the looks, stay for the personality? A mixed methods investigation of reacquisition and owner recommendation of Bulldogs, French Bulldogs and Pugs. PLoS ONE.

[47] Borgi M., Cirulli F. (2016). Pet face: Mechanisms underlying human-animal relationships. Front. Psychol..

[48] Bouma E.M.C., Vink L.M., Dijkstra A. (2020). Expectations versus reality: Long-term research on the dog-owner relationship. Animals.

[49] Fugazza C., Mongillo P., Marinelli L. (2017). Sex differences in dogs’ social learning of spatial information. Anim. Cogn..

[50] Duranton C., Rödel H.G., Bedossa T., Belkhir S. (2015). Inverse sex effects on performance of domestic dogs (*Canis familiaris*) in a repeated problem-solving task. J. Comp. Psychol..

[51] Mongillo P., Pitteri E., Candaten M., Marinelli L. (2016). Can attention be taught? Interspecific attention by dogs (*Canis familiaris*) performing obedience tasks. Appl. Anim. Behav. Sci..

[52] Prato-Previde E., Marshall-Pescini S., Valsecchi P. (2008). Is your choice my choice? The owners’ effect on pet dogs’ (*Canis lupus familiaris*) performance in a food choice task. Anim. Cogn..

[53] Scandurra A., Pinelli C., Fierro B., Di Cosmo A., D’Aniello B. (2020). Multimodal signaling in the visuo-acoustic mismatch paradigm: Similarities between dogs and children in the communicative approach. Anim. Cogn..

[54] Müller C.A., Mayer C., Dorrenberg S., Huber L., Range F. (2011). Female but not male dogs respond to a size constancy violation. Biol. Lett..

[55] Lazarowski L., Krichbaum S., Waggoner L.P., Katz J.S. (2020). The development of problem-solving abilities in a population of candidate detection dogs (*Canis familiaris*). Anim. Cogn..

[56] Rooijakkers E.F., Kaminski J., Call J. (2009). Comparing dogs and great apes in their ability to visually track object transpositions. Anim. Cogn..

[57] Kubinyi E., Turcsán B., Miklósi A. (2009). Dog and owner demographic characteristics and dog personality trait associations. Behav. Process..

[58] Scandurra A., Alterisio A., Di Cosmo A., D’Aniello B. (2018). Behavioral and perceptual differences between sexes in dogs: An overview. Animals.

[59] Miklósi A., Kubinyi E., Topal J., Gácsi M., Viranyi Z., Csanyi V. (2003). A simple reason for a big difference: Wolves do not look back at humans but dogs do. Curr. Biol..

[60] Hare B., Brown M., Williamson C., Tomasello M. (2002). The domestication of cognition in dogs. Science.

[61] Marshall-Pescini S., Passalacqua C., Barnard S., Valsecchi P., Prato-Previde E. (2009). Agility and search and rescue training differently affects pet dogs’ behaviour in socio-cognitive tasks. Behav. Process..

[62] Carballo F., Cavalli C.M., Gácsi M., Miklósi Á., Kubinyi E. (2020). Assistance and therapy dogs are better problem solvers than both trained and untrained family dogs. Front. Vet. Sci..

[63] Marshall-Pescini S., Valsecchi P., Petak I., Accorsi P.A., Previde E.P. (2008). Does training make you smarter? The effects of training on dogs’ performance (*Canis familiaris*) in a problem solving task. Behav. Process..

[64] Soproni K., Miklósi A., Topal J., Csanyi V. (2001). Comprehension of human communicative signs in pet dogs (*Canis familiaris*). J. Comp. Psychol..

[65] Udell M.A.R., Giglio R.F., Wynne C.D.L. (2008). Domestic dogs (*Canis familiaris*) use human gestures but not nonhuman tokens to find hidden food. J. Comp. Psychol..

[66] Leonardi R.J., Vick S.J., Dufour V. (2012). Waiting for more: The performance of domestic dogs (*Canis familiaris*) on exchange tasks. Anim. Cogn..

[67] Bhattacharjee D., Mandal S., Shit P., Varghese M.G., Vishnoi A., Bhadra A. (2020). Free-ranging dogs are capable of utilizing complex human pointing cues. Front. Psychol..

[68] Schmidjell T., Range F., Huber L., Virányi Z. (2012). Do owners have a clever hans effect on dogs? Results of a pointing study. Front. Psychol..

[69] Scandurra A., Alterisio A., Di Cosmo A., D’Ambrosio A., D’Aniello B. (2019). Ovariectomy impairs socio-cognitive functions in dogs. Animals.

[70] Persson M.E., Roth L.S., Johnsson M., Wright D., Jensen P. (2015). Human-directed social behaviour in dogs shows significant heritability. Genes Brain Behav..

[71] Proops L., Walton M., McComb K. (2010). The use of human-given cues by domestic horses, *Equus caballus*, during an object choice task. Anim. Behav..

[72] Berman P.W., Rane N.G., Bahow E. (1970). Age changes in children’s learning set with win-stay, lose-shift problems. Dev. Psychol..

[73] Kis A., Radics E., Bolló H., Topál J. (2023). Methodological comparison of cancellation versus two-way choice spatial attention tests in humans and dogs. Front. Vet. Sci..

[74] Bolló H., File B., Topál J., Kis A. (2023). Side bias behaviour in dogs shows parallels to the hemispatial neglect syndrome. Anim. Behav. Sci..

[75] Simon T., Guo K., Frasnelli E., Wilkinson A., Mills D.S. (2022). Testing of behavioural asymmetries as markers for brain lateralization of emotional states in pet dogs: A critical review. Neurosci. Biobehav. Rev..

[76] Rogers L.J. (2023). Knowledge of lateralized brain function can contribute to animal welfare. Vet. Sci..

[77] Mongillo P., Pitteri E., Carnier P., Gabai G., Adamelli S., Marinelli L. (2013). Does the attachment system towards owners change in aged dogs?. Physiol. Behav..

[78] Amano O., Mizobe K., Bando Y., Sakiyama K. (2012). Anatomy and histology of rodent and human major salivary glands. Acta Histochem. Cytochem..

[79] MacLean E.L., Gesquiere L.R., Gee N., Levy K., Martin W.L., Carter C.S. (2018). Validation of salivary oxytocin and vasopressin as biomarkers in domestic dogs. J. Neurosci. Methods.

[80] Powell L., Edwards K.M., Bauman A., Guastella A.J., Drayton B., Stamatakis E., McGreevy P. (2019). Canine endogenous oxytocin responses to dog-walking and affiliative human-dog interactions. Animals.

